# Surface site density and utilization of platinum group metal (PGM)-free Fe–NC and FeNi–NC electrocatalysts for the oxygen reduction reaction†

**DOI:** 10.1039/d0sc03280h

**Published:** 2020-10-13

**Authors:** Fang Luo, Stephan Wagner, Ichiro Onishi, Sören Selve, Shuang Li, Wen Ju, Huan Wang, Julian Steinberg, Arne Thomas, Ulrike I. Kramm, Peter Strasser

**Affiliations:** The Electrochemical Catalysis, Energy and Materials Science Laboratory, Department of Chemistry, Technische Universität Berlin Straße des 17. 10623 Berlin Germany pstrasser@tu-berlin.de; Department of Chemistry and Department of Materials and Earth Sciences, Catalysts and Electrocatalysts Group, Technical University of Darmstadt Otto-Berndt-Str. 3 64287 Darmstadt Germany kramm@ese.tu-darmstadt.de; JEOL Ltd 3-1-2 Musashino, Akishima Tokyo Japan; Technische Universität Berlin, Center for Electron Microscopy (ZELMI) Straße des 17. Juni 135 10623 Berlin Germany; Functional Materials, Department of Chemistry, Technical Universität Berlin Hardenbergstr. 40 Berlin 10623 Germany

## Abstract

Pyrolyzed iron-based platinum group metal (PGM)-free nitrogen-doped single site carbon catalysts (Fe–NC) are possible alternatives to platinum-based carbon catalysts for the oxygen reduction reaction (ORR). Bimetallic PGM-free M_1_M_2_–NC catalysts and their active sites, however, have been poorly studied to date. The present study explores the active accessible sites of mono- and bimetallic Fe–NC and FeNi–NC catalysts. Combining CO cryo chemisorption, X-ray absorption and ^57^Fe Mössbauer spectroscopy, we evaluate the number and chemical state of metal sites at the surface of the catalysts along with an estimate of their dispersion and utilization. Fe L_3,2_-edge X-ray adsorption spectra, Mössbauer spectra and CO desorption all suggested an essentially identical nature of Fe sites in both monometallic Fe–NC and bimetallic FeNi–NC; however, Ni blocks the formation of active sites during the pyrolysis and thus causes a sharp reduction in the accessible metal site density, while with only a minor direct participation as a catalytic site in the final catalyst. We also use the site density utilization factor, *ϕ*_SD_surface/bulk__, as a measure of the metal site dispersion in PGM-free ORR catalysts. *ϕ*_SD_surface/bulk__ enables a quantitative evaluation and comparison of distinct catalyst synthesis routes in terms of their ratio of accessible metal sites. It gives guidance for further optimization of the accessible site density of M–NC catalysts.

## Abstract 2

The gravimetric surface density and ORR catalytic turnover frequency of Fe–NC and Fe/Ni–NC catalysts were investigated. Both catalysts feature chemically identical Fe sites, but the presence of Ni lowered the gravimetric surface density of Fe sites.

## Graphical abstract

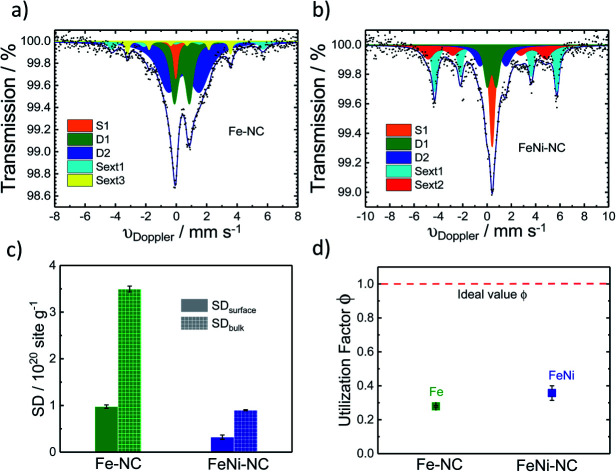

## 1. Introduction

The growing concerns about our current unsustainable energy infrastructure and its environmental effects have fueled research and development into alternative power supply technologies such as hydrogen fuel cells. Platinum-based catalysts have been identified as the most efficient catalysts for fuel cell cathodes and anodes. However, the high catalyst cost and their durability have remained hurdles for a wider commercialization of fuel cell technology. Carbon materials doped with non-noble transition metals and nitrogen, that is, PGM-free materials, are promising alternative catalysts for the electrochemical conversion of molecular oxygen; they already show promising performance in fuel cells, metal air batteries, and the chlor-alkali industry.^1–6^ The most important family of PGM-free ORR catalysts is the class of carbon-embedded, nitrogen-coordinated single metal site catalysts, typically referred to as metal–nitrogen–carbon (MNC) catalysts.^7,8^ Their state-of-the-art catalytic reactivity rivals that of state-of-the-art Pt nanoparticle catalysts.^1,9^ Particular attention was paid to a specific family of monometallic MNC catalysts derived from Co, Fe, and Mn in the active M–N_4_ moiety. Many studies confirmed their favorable catalytic activity for the ORR, as well as bimetallic M_1_M_2_–NC derived from them.^1,9–18^ Other PGM-free metals, such as Ni, and its bimetallic M_1_M_2_–NC ORR catalysts, however, have remained largely unexplored.^9,12,13,19–22^ Based on the design one tandem catalyst where combination a stronger O* binding sites on Fe and a weaker *OOH/*OH binding site on Ni,^23–28^ the co-existence of the atomic Fe and Ni single atom sites may results in an average chemisorption energy of ORR intermediates, as well as promising results of iron and nickel containing catalysts for the oxygen evolution reaction (OER), that it could be an interesting combination with potential promise for metal air battery applications and bifunctional ORR/OER capabilities.

Based on this, in this contribution, we analyze and contrast the fundamental reactivity of monometallic and bimetallic MNC ORR catalysts in previously unavailable ways. We address four distinct ORR catalysts: two mono-metallic Fe–NC and Ni–NC catalysts, the corresponding bimetallic FeNi–NC catalyst, and, as a reference, a “metal-free” (that is, metal content below the detection limit) NC catalyst. To study the effects of the combined presence of both metals on the physico–chemical and catalytic properties of these catalysts, we correlate their electrochemical ORR performance and X-ray absorption spectra (XAS) with their CO chemisorption and desorption behavior and their Mössbauer spectra. CO pulse chemisorption is a quite recent means to probe the total number of available metal active sites and thus yields conservative estimates of previously unavailable, intrinsic catalytic ORR turnover frequencies in alkaline and acidic electrolytes. Mössbauer analysis yielded the total site density in the bulk, from which a site utilization factor *ϕ*_SD_surface/bulk__ was derived and discussed.

## 2. Results and discussion

### 2.1. Synthesis, XPS, XAS and STEM analysis

Three MNC catalysts, a Fe–NC, a Ni–NC, and the corresponding bimetallic FeNi–NC ORR catalysts were synthesized using polyaniline (PANI) as a nitrogen precursor and metal salts as metal precursors. A metal-free N–C catalyst was prepared and served as a reference material. The catalyst precursors were subjected to a multi-step preparation involving pyrolysis steps (at 900 °C) and acid leaching steps.

**Fig. 1:**
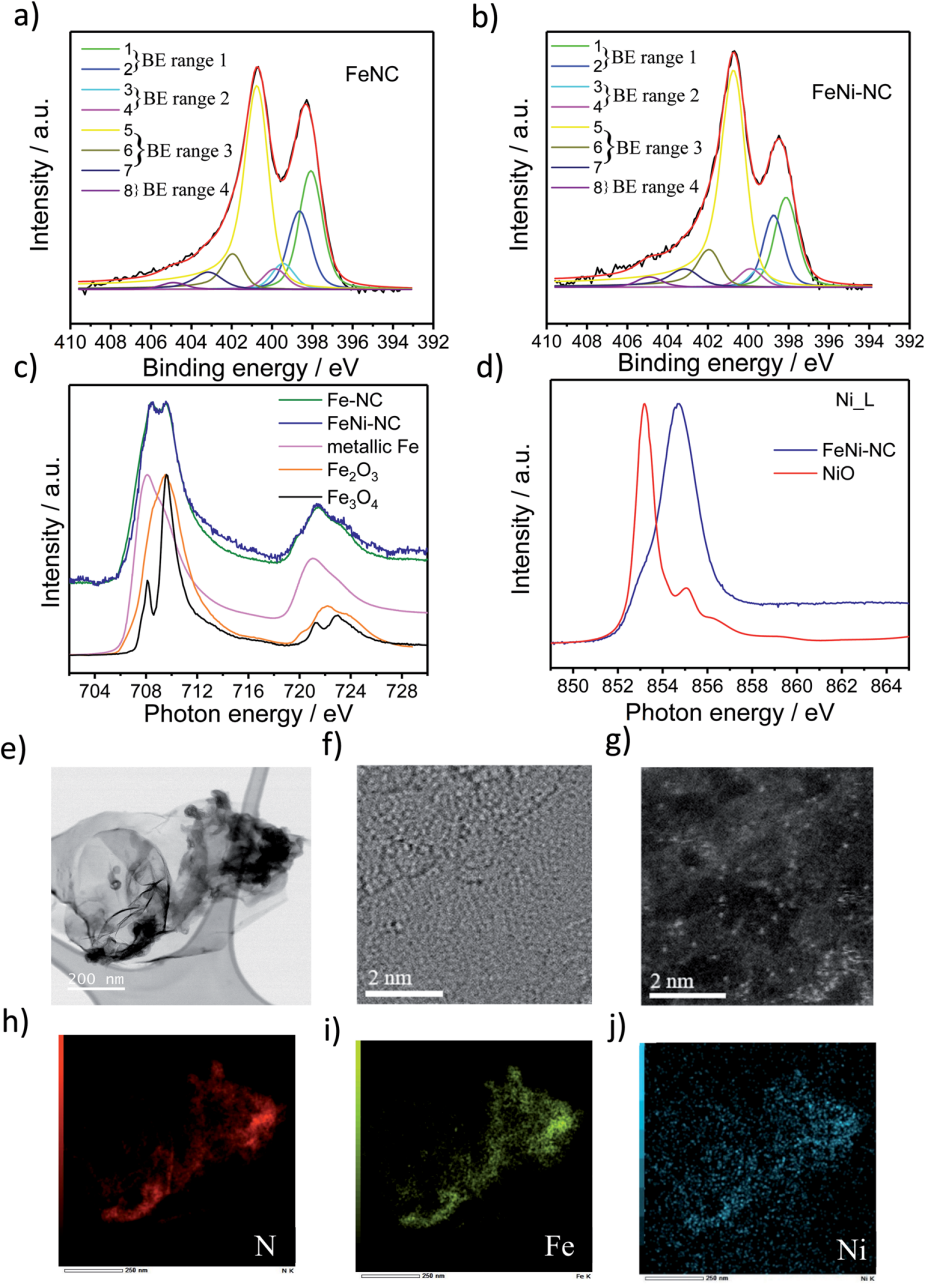
Characterization of the chemical states of N, Fe and Ni speciation using X-ray spectroscopy, low voltage probe-corrected scanning transmission electron microscopy (Cs-corr. STEM) and electron energy-loss spectroscopy (EELS) elemental maps. High-resolution N 1s XPS spectra of (a) Fe–NC and (b) FeNi–NC. The N1s XPS spectrum was fitted using eight individual spectral components that cover typical binding energy ranges of N species or motifs typically present in NC and MNC materials. The individual eight spectral component peaks were clustered into 4 BE-ranges. (c) Fe L_3,2_-edge spectra of catalysts and reference compounds. (d) Ni L-edge spectra of FeNi–NC and reference compounds. (e and f) Bright-field (BF) and high-resolution (HR)-STEM image of FeNi–NC catalysts. (g) High-angle annular dark-field (HAADF)-STEM image showing bright Fe and Ni atoms atomically dispersed in or on graphene layers. Element mapping image of the (h) N and (i) Fe and (j) Ni in FeNi–NC catalyst extracted from energy dispersive X-ray (EDX) spectroscopy, the mapping are related to (e).

To characterize the chemical state of the metal atoms and nitrogen atoms of the prepared catalysts, high-resolution X-ray photoelectron spectroscopy (XPS) and X-ray absorption spectroscopy (XAS) were used to investigate the N 1s region and the Fe and Ni L_3,2_ edge region (Fig. 1 and S1 and Table S1 and S2†), respectively. Sorption techniques were used to learn about the porosity characteristics of the catalysts (Table S3†). The N_1s_ XPS spectrum was fitted using eight individual spectral components that cover typical binding energy ranges of N species or motifs typically present in NC and MNC materials. The individual eight spectral component peaks were clustered into 4 BE-ranges. BE-range 1 includes Peak 1 (398.1 eV) and Peak 2 (398.7 eV) covering the N atom bonded to two sp^2^-hybridized carbons and –C=N–C double bond motifs (*e.g.* imine N, pyridinic N or triazinic N). BE-range 2 includes Peak 3 (399.3 eV) and Peak 4 (399.8 eV) covering the sp^2^ hybridized N atom in metal–N coordination, OC–NH–C partial double bonds, or multiple graphitic N motifs in a single aromatic ring (*e.g.* M–N_*x*_, amides). BE-range 3 includes Peak 5 (400.7 eV), Peak 6 (401.8 eV), and Peak 7 (403 eV) covering N atoms bonded with 1 H (in-plane hydrogenated N), isolated graphitic N, out-of-plane hydrogenated/protonated N and hydrogenated graphitic N (*e.g.* pyrrolic or protonated pyridinic and protonated graphitic N). BE-range 4 includes Peak 8 (405 eV) covering oxidized N (*e.g.* C=N–O or other oxidized N).^10,11,29–33^ The positions of the binding energies were chosen from DFT and experimental-DFT combinatorial studies on MNC and NC materials performed by Artyushkova *et al.*^9,28–30,33–37^ We note that this N-species assignment is unambiguous, yet a more accurate alternative evaluation of the relative contribution of N species is currently elusive,^33,38^ but requires probably an in-depth theoretical study that also considers the typical Fe/C ratios found in MNC catalysts. Our analysis in Fig. 1 and S1c and Table S2† suggested quite distinct relative concentrations of BE-range 2 in the PGM-free ORR catalysts: while the peak area is the lowest for FeNi–NC. Comparing the Fe and Ni L_3,2_-edge spectra of the Fe–NC and FeNi–NC catalysts (Fig. 1c and d) revealed further details about the chemical states of Fe and Ni.^39^Fig. 1c evidences Fe^2+^ and Fe^3+^ to be present in both Fe–NC and FeNi–NC, while the nitrogen-coordinated motif appeared to give rise to a characteristic “double resonance” feature in the 708 to 710 eV range. The presence of this feature indicated a very similar chemical state of the Fe–N_*x*_ sites in both catalysts. In FeNi–NC, there is no evidence of zero-valent Ni. Instead, the main absorption peak is shifted to higher photon energies and is assigned to a Ni^3+^ state with a minor Ni^2+^ state contribution (Fig. 1d).^39^ This was further supported by XPS (Fig. S1d and e†). The narrow-scan Ni 2p spectrum of FeNi–NC featured a broad peak in the 2p_3/2_ region at ∼854.2 eV, and a relatively weak satellite located at ∼861 eV indicating the Ni^2+^ oxidation state (Fig. S1d†). Difference spectra between the experimental FeNi–NC and expected envelopes of the Ni^2+^ compound based on the satellite feature suggested the presence of Ni^3+^, possibly in the carbon-embedded N-coordinated Ni–N_*x*_ format with axial oxygen ligands (Fig. S1e†).^28,40–46^

Carbon-hosted FeN_4_ single metal sites were recently directly imaged for the very first time using AC-STEM.^47^ The AC-STEM technique was used here to reveal the atomic-level structure and chemistry of a bimetallic FeNi–NC catalyst (Fig. 1e–j). The catalysts consisted primarily of fibrous carbon, graphene and amorphous carbon sheets (Fig. 1e, f and S2†). The dense fibrous carbon particles were composed of randomly oriented, intertwined, turbostratic graphitic domains on the order of a few nanometers in size (Fig. 1f, g and S2†). This indicated a very small graphitic domain size coupled with turbostratic graphene basal planes. Single metal atoms were dispersed homogeneously and randomly across the carbon sheets (Fig. 1g). The elemental mapping images by using EDX mapping with respect to N (red in Fig. 1), Fe (green), and Ni (blue) suggested that the Fe and Ni single metal sites were uniformly and homogeneously distributed over the entire carbon basal planes (Fig. 1h–j). The local co-existence of N and both Fe and Ni metal atoms provided evidence for the existence of coordinatively stabilized M–N_*x*_ motifs. The elemental mapping images thus confirmed our conclusions from XAS and XPS that single Fe and Ni metal atoms were successfully coordinated by N across the carbon domains. This is the direct observation of single atom catalysts with M–N_*x*_ type sites in bimetallic M_1_M_2_–NC catalysts, the N/M ratio could however not be quantified by STEM/EDX, as this resulted in the displacement of single heavy metal atom under an electronic beam, while the atomic ratio of Fe and Ni to N in FeNi–NC was, as expected, about the factor of 10, respectively (0.4 and 0.4 at% to 4.3 at% in Table S1,† and 1.74 wt%/56 and 2.44 wt%/59 to 4.3 wt%/14 in Table S3,† respectively).

To summarize this section, our XAS and XPS results revealed that the nitrogen-coordinated Fe-centers in Fe–NC and FeNi–NC appear chemically identical. However, the site density of BE-range 2 moieties in the bimetallic FeNi–NC catalyst appeared to have decreased sharply upon introducing Ni as a second metal (Fig. S1c†). As we will show below, this has consequences on the electrocatalytic reactivity.

### 2.2. Electrocatalytic oxygen reduction activity

**Fig. 2:**
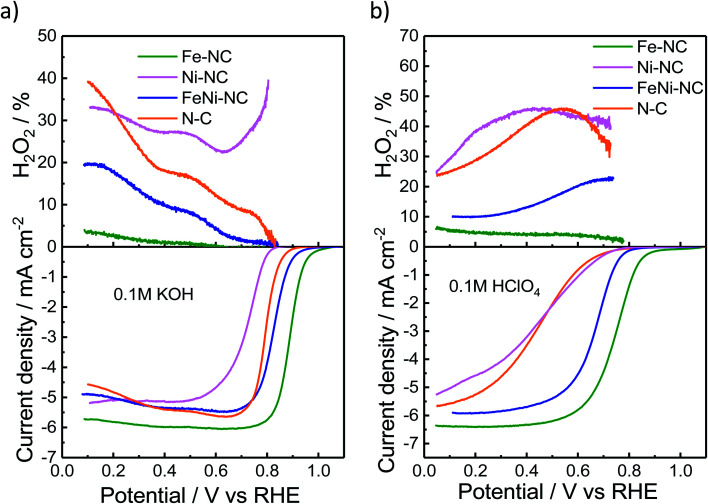
Electrochemical activity evaluation. H_2_O_2_ percentage and disk current density from rotating-ring disk electrode (RRDE) experiments of MNC catalysts at 1600 rpm in oxygen-saturated (a) alkaline and (b) acidic electrolytes.

We used the rotating-ring disk electrode (RRDE) technique to obtain the catalytic oxygen reduction reactivity in alkaline and acidic electrolytes. Fig. 2a and b display the measured linear sweep voltammograms during the ORR, characterized by their kinetic, mixed, and diffusion-limited regions (<∼0.4 V_RHE_). The number of transferred electrons (Fig. S3†) is a measure of the ratio of the direct 4e^−^ reduction and the 2e^−^ peroxide pathway^48^ for each catalyst. The highest ORR activity was observed for Fe–NC in either pH range. Fe–NC showed around 50 mV and 90 mV lower over-potentials (at 0.5 mA cm^−2^) than FeNi–NC in KOH and HClO_4_, respectively. FeNi–NC and the N–C reference showed comparable activities in KOH, while, in the acid electrolyte, the N–C reference catalyst showed a sharply lower ORR activity (increased overpotential, see Fig. 2b). Fe–NC showed favorable, small H_2_O_2_ yields of 4% in alkaline and 8% in acid electrolytes (at 0.1 V_RHE_). FeNi–NC showed a clearly higher H_2_O_2_ yield, followed by the N–C catalyst. Despite a comparable total (Fe + Ni) metal content of about 4 wt% (Table S3†) for both the Fe–NC and the FeNi–NC catalysts, the bi-metallic FeNi–NC catalyst showed a significantly lower catalyst mass-based ORR activity (Table S4†). We attribute this to the sharply reduced Fe wt% in the bimetallic catalyst. Considering the identical synthesis procedure and conditions, we are inclined to conclude that the competition between Ni and Fe metal sites during the bimetallic FeNi–NC catalyst formation process resulted in fewer Fe–N_*x*_ sites and a lower overall Fe concentration in the surface and bulk (Tables S1–S3†). Indeed, the calculated energy of formation of Ni–N_4_ sites is lower and thus more favorable in comparison to that of Fe–N_*x*_.^49^ In addition, we hypothesize that the generated Ni–N_*x*_ moieties exhibit a lower intrinsic ORR reactivity than Fe–N_*x*_ sites. This hypothesis is indeed supported by studies of MNC ORR catalysts.^17,50,51^ In that study, Zagal reported that the Fe(iii)/Fe(ii) redox process provides the driving force for the catalytic ORR thanks to the nearly half-filled d-energy levels of Fe; in contrast, such d characteristics are lost in metals such as Cu and Ni.^46^ Also, Fig. 2 directly confirms our hypothesis that Ni–NC sites display a very low catalytic ORR activity; under alkaline conditions, even worse than the N–C reference catalyst. Among the MNC materials, the Ni–NC catalyst showed the lowest ORR activity in both acid and alkaline electrolytes. Its catalyst mass activity, *j*_m_, dropped by a factor of ∼85× in the acid compared to that of Fe–NC, while its half wave potential was up to 340 mV higher than that of Fe–NC (Tables S4 and S5†).

**Fig. 3:**
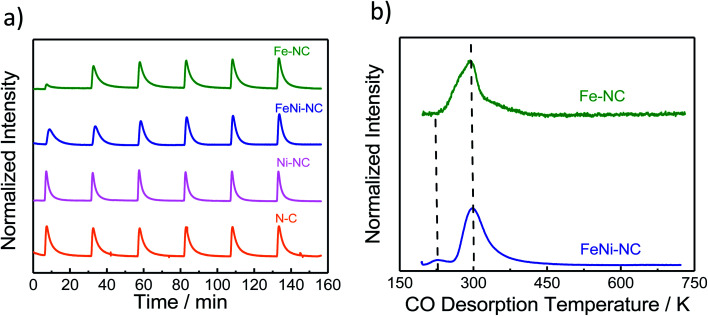
The molar CO uptake and TPD results. (a) CO pulse chemisorption and (b) normalized temperature-programmed desorption (TPD) profiles of MNC catalysts.

The electrochemical Tafel curves in Fig. S3c and d† illustrate the effect of the applied electrode potential on the observed catalytic ORR current density. In an ideal one-electron transfer approximation, the Tafel slope is viewed as a measure of the kinetic exponent of the electrons in the kinetic rate law derived from the rate-determining elementary step. However, in more realistic multi-step reaction mechanisms that include bond making and bond breaking between adsorbed intermediates, such as in the ORR, the Tafel slope becomes a function of many different factors, such as the potential-dependent coverage of oxygenated intermediates. The potential dependence of the surface intermediate coverage may follow a simple Langmuir, a Temkin, or, in more complex cases, even a Frumkin isotherm.^52^ As such, the Tafel slope may vary dramatically with applied potential, even though the rate-determining step remains the same. This is why an interpretation of the Tafel slopes in terms of a simple sequence of chemical or electrochemical elementary steps is mostly difficult, and this is why we refrain from an oversimplified interpretation of the Tafel slope values.^53^ That said, Fig. S3c and d† reveal low Tafel slopes of 57 and 85 mV dec^−1^ for Fe–NC in KOH and HClO_4_, respectively. The FeNi–NC catalyst showed rather similar Tafel slopes of 68 and 87 mV dec^−1^ in KOH and HClO_4_, respectively. Here, it may be safe to assume that the ORR rate-determining step remained unchanged under either pH conditions. A remarkable difference in the Tafel slope was observed for the N–C reference catalysts in the acid compared to alkaline media (*ca.* 160 mV dec^−1^*vs. ca.* 61 mV dec^−1^). This is in line with earlier reports on the distinctly different purely chemical reaction step between the first and second electron transfer step.^53^ Fe–NC displayed an electron transfer number of close to 4, while N–C showed the lowest electron transfer number coupled with the highest H_2_O_2_ yield during the ORR (Fig. S3†). According to a recent model of the ORR pathway (Fig. 3 and S4 in ref. 53†), together with the results regarding the selectivity, electron transfer number and Tafel slopes, the 4e^−^ ORR pathway is most favorable on the Fe–NC catalyst, while the FeNi–NC catalyst appears to support a mixture of 2e^−^ and 4e^−^ pathways, in accordance with its mixed Fe–N_*x*_ and Ni–N_*x*_ sites, while the N–C and Ni–NC catalysts are prone to an almost pure 2e^−^ oxygen reduction pathway.

### 2.3. CO cryo-adsorption and temperature-programmed desorption (TPD)

Carbon monoxide (CO) has long been proposed as a blocking probe molecule of the catalytically active sites of FeNC ORR electrocatalysts, because CO can form strong metal–ligand coordination bonds.^54,55^ As such, it would inhibit adsorption and reaction on accessible metal sites that catalyze the oxygen reduction reaction.^55–57^ However, CO adsorption and stripping under electrochemical conditions have remained elusive. Recently, however, it was reported that CO cryo adsorption on accessible FeN_*x*_ sites of pyrolyzed Fe–N–C ORR catalysts at temperatures at around −80 °C following careful thermal annealing protocols does, in fact, allow an accurate quantification of the gas-accessible Fe–N_*x*_ sites on the surface and hence an evaluation of the accessible metal site density.^9,58,59^Fig. 3a shows the adsorption trajectory of six consecutive CO adsorption pulses for each MNC catalyst. The gradual increase in the apparent CO pulse area indicated CO adsorption by the accessible metal sites of the catalyst until full saturation levels were reached in the 4^th^ or 5^th^ pulse. While Fe–NC showed the largest CO uptake, FeNi–NC showed clearly reduced CO uptake (reduced by a factor around three). In contrast, no measureable adsorption of CO was detected on the N–C reference or the Ni–NC ORR catalyst, despite the highest abundance of Ni–N_*x*_ moieties. Clearly, the nature and the abundance of the metal centers play a key role in the CO pulse chemisorption process for MNC ORR catalysts.^40,41^ Ni–N_*x*_ sites are unable to bind CO strongly enough, even at low temperatures.

**Fig. 4:**
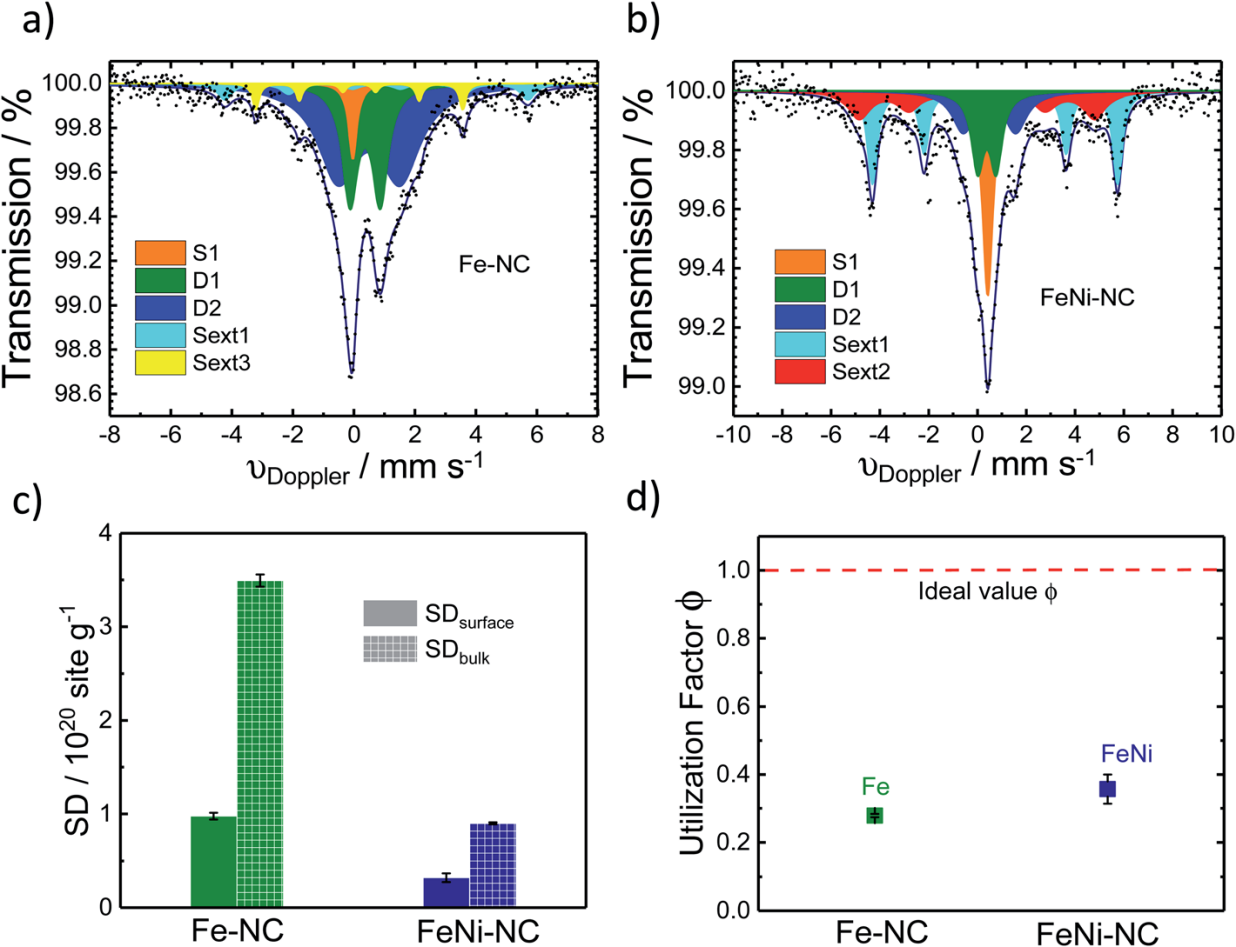
^57^Fe Mössbauer spectra of (a) Fe–NC and (b) FeNi–NC catalysts. (c) Comparison of SD_surfacce_ and SD_bulk_ derived from CO cryo chemisorption and Mössbauer spectroscopy experiments (all FeN_4_ species, D1 + D2), respectively. (d) The active-site utilization factor *ϕ*_SD_surface/bulk__ as defined in eqn (3) is plotted for the Fe–NC and FeNi–NC, and the red dotted line means ideal utilization*ϕ*_SD_surface/bulk__ = 1.

The molar CO uptake of the Fe–NC and the FeNi–NC catalysts was converted into a catalyst mass-based accessible site density on the surface, SD_surface_, with the unit site g^−1^ (Table S6†). Fig. 4 (solid bars) compares the resulting SD_surface_ values of the monometallic Fe–NC and the FeNi–NC catalysts.

The sharply lower CO uptake for the Ni-containing bi-metallic sample is consistent with the lower electrocatalytic ORR reactivity of the FeNi–NC catalysts and is in excellent agreement with our physico-chemical analysis, which suggested a reduced Fe content in the bimetallic catalyst. The presence of Ni precursors, combined with the rapid formation of Ni–N_*x*_ sites at the surface limits or partially suppresses the generation of Fe–N_*x*_ active sites in FeNi–NC, resulting in lower CO chemisorption. This is the first time of an experimental confirmation of the prediction already made by theory (compare Fig. S7 in ref. 49†). Moreover, the Ni–NC catalyst showed the lowest ORR activity, which, judging from its CO uptake, may be due to the insufficiently strong bond between the Ni central atom and reactive oxygenated intermediates. This is why Ni–N_*x*_ is unable to split the O–O bond and favors the 2e^−^ oxygen reduction pathway to H_2_O_2_.

As shown in Fig. 3b, following the CO cryo uptake, both the Fe–NC and the FeNi–NC catalysts showed a major CO desorption peak around 300 K. However, the shape of Fe–NC also indicates an additional contribution probably from a second-type of Fe–N_*x*_ site. This shoulder is not as intensive for FeNi–NC, while the main peak associated with most of the FeN_*x*_ sites is identical. This may suggest similar CO bond strengths and desorption rates from the CO-adsorbing Fe–N_*x*_ sites, and ultimately a similar nature of the adsorbing Fe–N_*x*_ sites of these two catalysts. This finding is in line with our results from XAS (Fig. 1c) where the Fe L_3,2_-edge spectra suggested a close chemical similarity between both catalysts. However, the number of active Fe–N_*x*_ sites, that is, the active site density, was quite different for the two catalysts, as unambiguously revealed by the CO chemisorption experiments. Considering the identical initial molar amounts of Fe precursors at the beginning of the synthesis and the identical synthesis procedures, we are inclined to conclude that the bimetallic FeNi–NC catalysts displayed fewer active Fe–N_*x*_ surface sites due to the presence of Ni as a second metal precursor. We speculate that the Ni precursor affects the formation rate of active Fe–N_*x*_ during the pyrolysis process, or else affects the dispersion of the Fe–N_*x*_ sites of the resulting catalyst material.^60^

In summary, the data above suggest that the CO adsorption and CO desorption are strongly governed by the chemical nature and accessible density of the N/C-coordinated central metal ion site. Fe-sites show strong uptake, while Ni-sites show weak to none. The FeNi–NC catalyst displayed a sharply reduced CO uptake which is in agreement with the significantly smaller content of nitrogen associated with Fe–N_*x*_ sites. The presence of Ni during the synthesis and Ni–N_*x*_ centers implanted to this catalyst appears to negatively impact the formation of catalytic active Fe–N_*x*_ sites on the surface, which accounts for the reduced CO uptake.

### 2.4. The active site density in bulk and the utilization factor

^57^Fe Mössbauer spectroscopy was used to estimate the number of Fe–N_*x*_ sites of the catalyst (Fig. 4a and b). It is a powerful fingerprinting technique to determine different electronic and chemical iron states.^61^

In the Fe–NC sample five different iron species can be identified (Fig. 4a). Beside a superparamagnetic iron phase (S1, *δ*_iso_ = −0.04 mm s^−1^)^62^ two doublets and two singlets were assigned. The doublet D1 (*δ*_iso_ = 0.37 mm s^−1^, Δ*E*_Q_ = 0.99 mm s^−1^) is found for all Fe–NC catalysts investigated by Mössbauer spectroscopy so far^62–69^ and represents a ferrous FeN_4_ site in the low-spin state. As discussed by Kramm *et al.*, its overall coordination environment is most likely six-fold with a nitrogen ligand and the oxygen molecule in axial directions.^60,61,70,71^ This site was previously assigned as the ORR active site by us and others;^63,68,72–74^ however it should be noted that small iron oxide nanoparticles can also lead to a Mössbauer doublet with similar parameters.^71,75^ The doublet D2 (*δ*_iso_ = 0.50 mm s^−1^, Δ*E*_Q_ = 2.04 mm s^−1^) shows an isomer shift with a ferrous intermediate spin site. However, the quadrupole splitting is almost in-between the values found for iron porphyrins and iron phthalocyanine (FePc).^61^ The large quadrupole splitting for FePc in relation to iron porphyrins is caused by a pseudo-six fold coordination of the FeN_4_ sites with the above and underlining aza-bridges of the stacked FePcs.^76^ Based on this, the intermediate quadrupole splitting observed for the D2 doublet in this work might still be caused by an interaction in the axial direction, which is possibly not as much pronounced in relation to that of the phthalocyanine. The catalyst further showed two inorganic iron components assigned as iron sulphide (sext1, *δ*_iso_ = 0.74 mm s^−1^)^77^ and iron carbide (sext3, *δ*_iso_ = 0.18 mm s^−1^).^78^ Both phases are formed frequently during the heat treatment of iron based catalysts.^77^ The relative absorption areas are given in Table 1. Following previous studies, we assume the D1 site to be the primary catalytically active site of our catalysts.^68,73,79^ The overall amount of inorganic iron species amounts to less than 20%, whereas more than 80% of the Fe atoms are in fourfold coordination with nitrogen atoms.

**Table 1:** Mössbauer parameters for iron sites as found in Fe–NC and FeNi–NC

	*δ*/mm s^−1^	Δ*E*_Q_/mm s^−1^	*H*_0_/T	fwhm/mm s^−1^	Rel. area/%
**Fe–NC**					
D1	0.37	0.99		0.61	27.4
D2	0.50	2.04		1.59	55.5
Singlet1	−0.04			0.34	4.9
Sext1	0.74		30.86	0.58	7.0
Sext3	0.18		21.01	0.27	5.2

**FeNi–NC**					
D1	0.39	0.75		0.61	15.8
D2	0.50	2.15		1.1	18.5
Singlet1	0.42			0.52	15.1
Sext1	0.72		31.22	0.47	30.2
Sext2	−0.02		29.98	1.09	20.4

For the nickel-containing FeNi–NC catalyst sample (Fig. 4b) a different behavior was observed. The Mössbauer spectral fit identified a singlet with an isomer shift (*δ*_iso_ = 0.42 mm s^−1^) that is unusual for paramagnetic iron phases. We are not able to assign this singlet now to a known species only based on Mössbauer parameters. Low-temperature Mössbauer spectroscopy might be used in the future to give further insights. As for the “sext2” species a mere Mössbauer parameter-based assignment to both alpha-Fe and a FeNi alloy with 30% Ni is possible.^80–83^ Beside these inorganic phases, the sample FeNi–NC contains the mentioned D1 and D2 sites with absorption areas of 15.8% and 18.5%, respectively. The experimentally determined trend in the abundance of the D1 sites (Table 1) agrees well with that in the electrocatalytic activity of Fe–NC and FeNi–NC (note: beside a larger relative fraction in terms of absorption the iron content in FeNi–NC is also lower). The higher abundance of the catalytically active D1 sites in the Fe–NC catalyst (Table 1) is responsible for the higher electrochemical activity of the Fe–NC catalyst and is consistent with the trends in the Fe–N_*x*_ site bulk density (SD_bulk_) values discussed below. Hence, while FeNi–NC features active Fe–N_*x*_ sites at the surface with a nearly identical chemical nature, its Fe–N_*x*_ surface site density is lower than that of the Fe–NC catalyst and it contains more inorganic Fe-based phases. We consider the larger formation of inorganic Fe-based phases in the FeNi–NC catalyst compared to Fe–NC to be the consequence of the presence and competing reactivity of the Ni precursor during the synthesis.

As the TPD data indicate that not only one single type of FeN_*x*_ site adsorbs the CO, for the bulk-related site density we considered the overall iron assigned to the doublets D1 and D2. However, as recently shown, other ORR inactive iron speciation might overlay in the central part of the Mössbauer spectrum, so D1 and D2 might be overestimated.^71^ This must be considered and taken into account. Thus, the value determined from Mössbauer spectroscopy needs to be considered as the upper limit of possibly present FeN_*x*_ sites.

Active site utilization factors provide useful guidelines for a directed synthesis of catalyst materials with optimized active site dispersion on the surface. Surface site utilization factors are measures for the ratio of the total number of Fe–N_*x*_ sites that are located on the surface of the catalyst. Efficient catalysts are characterized by large site utilization factors. For example, in our previous work, the utilization factor was strongly dependent on the number of heat-treatment and preparation steps.^9^ For the purpose of evaluating surface site utilization factors, we point out the fundamental difference between the CO chemisorption experiments and the Mössbauer spectroscopy. CO chemisorption is a method to quantify the accessible Fe–N_*x*_ metal sites on the surface. In contrast, Mössbauer spectroscopy is a bulk method probing the maximum density of the active Fe–N_*x*_ sites. For a catalyst where all active sites are surface accessible the number of sites identified by CO sorption should be identical to the number from Mössbauer spectroscopy.

From the Mössbauer parameters the catalyst mass-based Fe–N_*x*_ site density in the catalyst bulk, SD_bulk_, was derived and is plotted in Fig. 4c (hatched bars). For the calculation of SD_bulk_ both doublets D1 and D2 were considered, thus reflecting the maximum possible number of FeN_*x*_ sites^9,58,81,84,85^ and it was evaluated using the following relation:1
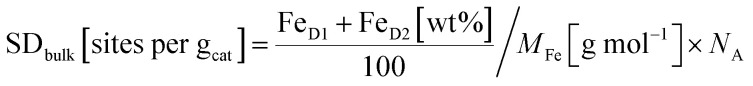
where *M*_Fe_ represents the molecular mass of iron and *N*_A_ is Avogadro's number. Fig. 4c evidences that the SD_bulk_ value (hatched bars) of Fe–NC amounts to 3.49 × 10^20^ sites per g and, thus, is about 4× larger than that of the FeNi–NC catalyst (0.90 × 10^20^ sites per g).

The CO-adsorption data were used to quantify the catalytically active adsorption sites on the catalyst surface. SD_surface_, on the other hand, was estimated according to:

2 SD_surface_ [sites per g_cat_] = *n*_CO_ [mol g^−1^] × *N*_A_

where *n*_CO_ represents the molar CO uptake, and *N*_A_ is Avogadro's number. The molar amount of adsorbed CO per mg catalyst, *n*_CO_ (mol g^−1^), equals the molar concentration of Fe–N_*x*_ surface sites, assumed to contribute to the ORR process.

Fig. 4c shows that both SD_surface_ and SD_bulk_ of the Fe–NC catalyst are significantly larger than those of FeNi–NC (Table S7†), which is in accordance with the observed trends in electrocatalytic ORR reactivity and our conclusions above.

Combining the SD_surface_ and SD_bulk_ values, a site utilization factor can be derived according to:^9^

3 *ϕ*_SD_surface/bulk__ = SD_surface_/SD_bulk_

*ϕ*_SD_surface/bulk__ represents the fraction of the total number of Fe–N_*x*_ sites in the catalyst that are located in the surface and are accessible to CO cryo adsorption (Table S8†). Fig. 4d compares the utilization factors *ϕ*_SD_surface/bulk__ of the two catalysts. The red dashed line represents *ϕ*_SD_surface/bulk__ = 1, that is, the ideal situation where all single metal Fe–N_*x*_ sites are located at the surface and hence are CO accessible. Interestingly, even though the absolute number of metal sites differs sharply, the utilization factors *ϕ*_SD_surface/bulk__ of FeNi–NC and Fe–NC are very similar, ranging around 33% (Tables S7 and S8†). This raises an important question: Given that the generation rate of metal sites during the synthesis differs in the presence of Ni, did the utilization factors turn out so similar due to comparable surface area/porosity characteristics of the initial carbon precursors or of the resulting carbon catalysts? To check the hypothesis whether the utilization factor of a MNC catalyst is controlled by surface area or porosity,^58^ Fig. S4† correlates the utilization factors with the BET surface areas of the two catalysts (Tables S3 and S8†). The FeNi–NC catalyst displayed a clearly larger BET surface area evidencing that BET values do not significantly affect the site utilization factors (Fig. S4†). Similarly, the correlation of the micro and mesopore volumes and the utilization factors is shown in Fig. S5a and b.† Neither micro nor mesopore volume appears to significantly control the resulting site utilization factors. The correlations of Fig. S4 and S5† suggest that the Fe–N_*x*_ sites are generated uniformly and are distributed uniformly across the bulk and surface of the catalysts. The presence of Ni, however, caused a larger BET surface area and larger micropore volume, likely due to catalytic carbon etching processes caused by Ni atoms during the high temperature pyrolysis.

The general importance of the site utilization factor *ϕ*_SD_surface/bulk__ lies in its guiding nature for the synthetic optimization of the surface site density (SD_surface_). An optimized synthesis generates MNC catalysts exhibiting *ϕ*_SD_surface/bulk__ = 1, that is, all Fe–N_*x*_ sites are located at the surface and can potentially contribute to the surface catalytic reaction process. Comparison of *ϕ*_SD_surface/bulk__ values obtained from varying synthesis pathways allows the selection and prioritization of synthesis pathways and identification of synthetic variables that favor dispersion of sites on the surface. Once *ϕ*_SD_surface/bulk__ = 1, the synthesis pathway is optimal, and further efforts to improve the catalytic reactivity can focus on improving the intrinsic turnover frequency.

### 2.5. Catalytic turnover frequency (TOF)

The TOF is an intrinsic reactivity descriptor of a catalyst. It describes the number of catalytic turnover events per catalytically active site and second. In electrochemistry, TOF values are often normalized to the number of electrons transferred per active site and second.

To evaluate electrocatalytic TOF values, RRDE-derived, mass-transport corrected, kinetic current densities are normalized by the number of catalytic surface sites. Here, we estimate mean TOF values from the molar CO uptake, *n*_CO_, and the kinetic catalyst mass-based current density, *j*_m_, evaluated at 0.85 V *vs.* RHE in alkaline and 0.8 V *vs.* RHE in acidic media.

The CO-derived TOF values, TOF, were calculated using the relation:

4 TOF [e^−^ s^−1^ site^−1^] = *j*_m_ [e^−^ s^−1^ g^−1^]/*n*_CO_ [site g^−1^]

**Fig. 5:**
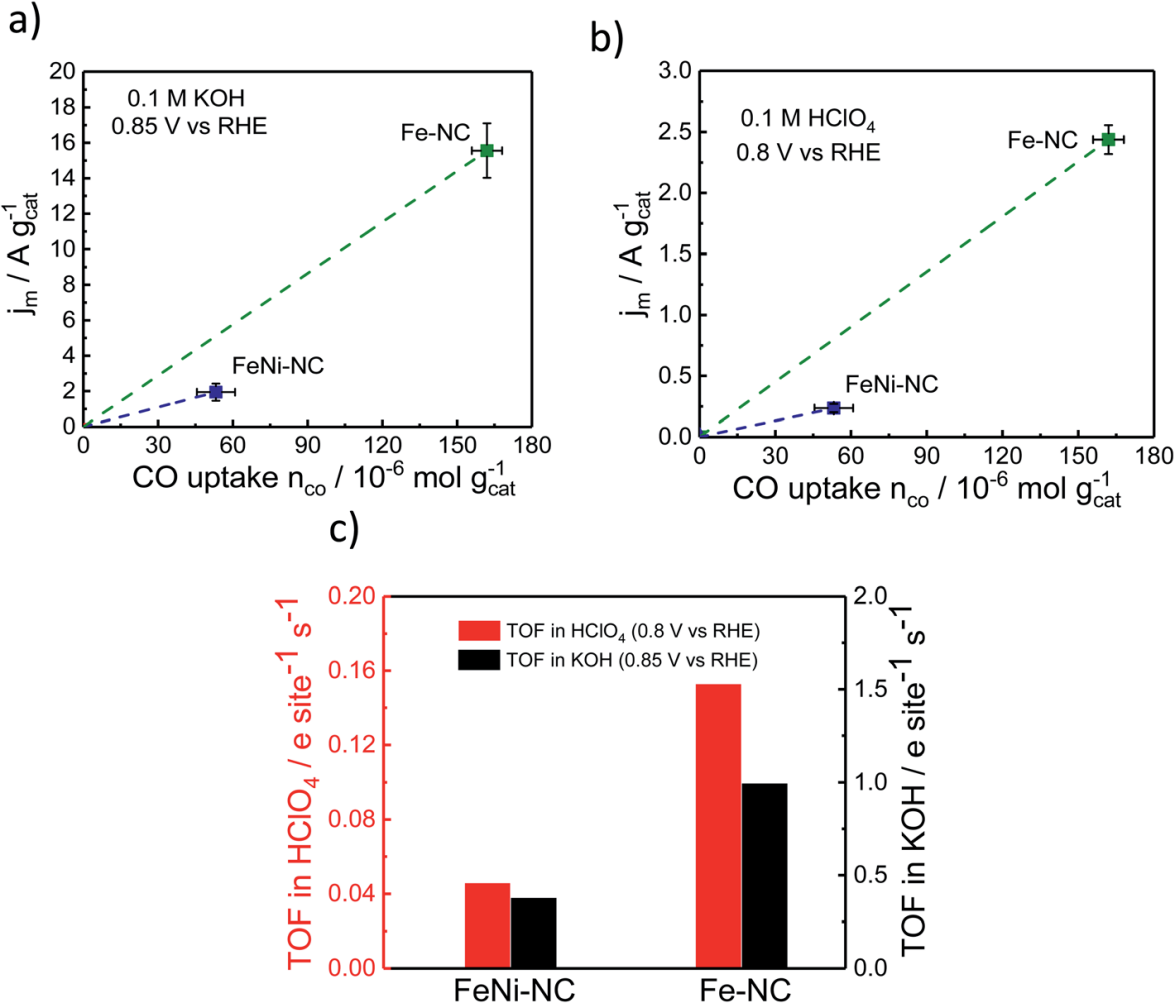
ORR catalyst kinetic mass activity *j*_m_ at (a) 0.85 V *vs.* RHE in alkaline and (b) 0.8 V *vs.* RHE in acidic media, respectively as a function of CO uptake. (c) Comparison of the turn-over frequency values as derived from CO chemisorption and RRDE experiments in acid and alkaline electrolytes.

Fig. 5 displays the relevant *n*_CO_*vs. J*_m_ relation, the slopes of which are the respective TOF values. The catalytic TOF values of the Fe–NC catalyst were ∼3× larger than those of the bimetallic FeNi–NC catalyst for both acid and alkaline electrolytes (Table S9†). This is mainly because the kinetic catalyst mass-based ORR activity, *j*_m_ (at 0.85 V_RHE_ in KOH and 0.8 V_RHE_ in HClO_4_, respectively) of FeNi–NC was markedly smaller, in particular by a factor of around 8×. This sharp drop in kinetic activity was not offset by the roughly 3× smaller SD_surface_ value of the FeNi–NC catalyst, which is why bimetallic FeNi–NC appeared as an intrinsically less active ORR catalyst compared to the Fe–NC catalyst (Fig. 5c). This seems at odds with the discussion above: based on our analysis of the chemical state of the Fe–N_*x*_ sites in Fe–NC and FeNi–NC, we concluded that both Fe–N_*x*_ sites display very similar physico-chemical characteristics; hence, we would expect similar intrinsic catalytic TOF values, regardless of the varying absolute number of active sites at the surface (SD_surface_). There are different reasons that could contribute to the observed discrepancy between the TOF values of Fe–NC and FeNi–NC. Firstly, we point to the larger ratio of micropores in the bimetallic FeNi–NC catalyst (Table S3†). While active Fe–N_*x*_ sites on the inner walls of micropores are probed by the CO cryo chemisorption technique, electrolyte flooding combined with diffusional oxygen transport limitations and ionomer poisoning could lower the apparent catalyst mass-based ORR activity of the FeNi–NC catalyst (see Fig. 5a and b). Secondly, the ORR mechanism and rate-limiting step could be different during the ORR for FeNi–NC compared to Fe–NC (Fig. 2 and S3†). The higher H_2_O_2_ yields of the FeNi–NC catalyst appear to support the co-existence of a 2e^−^ and 4e^−^ pathway, where the enhanced exposure of FeNi–NC to H_2_O_2_ may affect the reactivity of the catalytic sites.^53^ Thirdly, it was shown before that additional nitrogen atoms in the carbon frame can enhance the TOF value.^69^ As indicated in Tables S1 and S3† the nitrogen content of Fe–NC is much higher compared to that of FeNi–NC. Thus, a larger contribution of nitrogen functional groups and consequently TOF-increasing effects is likely for Fe–NC. While we cannot discriminate the individual contributions of these possibilities, as a result, the slopes of the FeNi–NC catalyst and the TOF values are smaller than those of Fe–NC (Fig. 5c).

## 3. Conclusions

The objective of the present study was to achieve a deeper understanding of the chemical nature, surface site density, and intrinsic reactivity (turnover numbers) of the atomically dispersed, catalytically active Fe–N_*x*_ sites located at the surface of a bimetallic PGM-free FeNi/N-doped carbon (“FeNi–NC”) ORR catalyst and of the monometallic Fe–NC and Ni–NC analogues. We provided evidence that Fe–N_*x*_ sites and not Ni–N_*x*_ sites are responsible for the kinetic ORR reactivity. XAS and Mössbauer spectroscopy as well as CO desorption data suggested that the chemical nature and bonding characteristics of the Fe–N_*x*_ sites are very similar for the Fe–NC and FeNi–NC catalysts. CO cryo chemisorption experiments and Mössbauer spectroscopy, on the other hand, yielded an estimate of the metal Fe–N_*x*_ site densities in the surface (SD_surface_) and in the bulk (SD_bulk_) of Fe–NC and FeNi–NC. Contrasting these site density values for each catalyst and between the two catalysts, we concluded that the co-generation and/or presence of Ni–N_*x*_ sites in the bimetallic catalyst partially suppressed the generation of catalytically active Fe–N_*x*_ sites, which, in turn, reduced the resulting SD values of FeNi–NC by a factor of 3. The reduction in the corresponding kinetic ORR reactivity of the bimetallic FeNi–NC catalyst, however, sharply exceeded this factor (8×) and might have different origins as changes in the contribution of pore regimes and the higher exposure of active sites to H_2_O_2_ could affect their reactivity or higher intrinsic TOF of Fe–NC by nitrogen doping of the graphene planes. As a result, the intrinsic TOF values of FeNi–NC fell short of those of Fe–NC, despite chemical identity.

The site density values further allowed the estimation of a site utilization factor *ϕ*_SD_surface/bulk__. *ϕ*_SD_surface/bulk__ offers rational guidelines for the design and synthesis of PGM-free M–NC catalysts toward optimal metal site dispersion on the surface. By means of the *ϕ*_SD_surface/bulk__, catalyst development can address the increase in the dispersion of catalytic surface sites and the increase in the catalytic TOF values independently.

## 4. Experimental methods

### 4.1. Catalyst synthesis

Aniline (Sigma-Aldrich) was first dissolved in 0.5 M HCl solution. Thereafter, metal precursors (6.05 g FeCl_3_·6H_2_O for Fe–NC, 6.05 g FeCl_3_·6H_2_O and 1.26 g NiCl_2_·6H_2_O for FeNi–NC, and 2.60 g NiCl_2_·6H_2_O for Ni–NC) were added into the solution and kept stirring for 15 min to be fully mixed with aniline. After the complete dissolution/mixing of the precursor with aniline, ammonium peroxydisulfate (APS, (NH_4_)_2_S_2_O_8_, Sigma-Aldrich) was dissolved in the solution as an oxidant to form poly-aniline, followed by adding activated carbon which was pretreated with 0.5 M HCl and 67% HNO_3_. The polymer suspension was kept at room temperature as long as no color change was observed. Carbon supported metal/poly-aniline was refluxed for 5 hours at 80–90 °C and then dried at 80 °C overnight by using an oil bath. The subsequently dried powder was ball milled and pyrolyzed at 900 °C for 1 hour in N_2_ (blowing N_2_ through the quartz tube at room temperature for 2 hours, then 5 °C min^−1^ temperature ramp for heating up to 900 °C, holding 1 hour under N_2_ and subsequent cooling overnight under N_2_). After the first pyrolysis step, one typical acid leaching step was used to remove all unreacted residues: the well-ground powder was dispersed in a diluted acidic solution and refluxed under a N_2_ atmosphere at 90 °C for 8 hours. After acid leaching, the samples were washed to neutralize them and then dried. The acid leached powders went through a second heat-treatment at 900 °C for 1 hour (2nd pyrolysis) followed by a second acid leaching/washing/drying step. Finally, the third pyrolysis (900 °C/1 h/N_2_) step was performed to achieve the final catalyst.

### 4.2. Physical characterization

Elemental analysis for determining the catalysts' composition was performed by using a Thermo Electron, Flash EA 1112 analyzer. The N_2_-physisorption measurements were performed on an Autosorb-1-C from the Quantachrome Company. The samples were first degassed by heating for 24 hours at 60 °C in a vacuum. Before the Brunauer–Emmett–Teller (BET) measurements, the samples were cooled using a flow of liquid nitrogen and continuously loaded with nitrogen during the measurements. After saturation of N_2_ on the catalyst surface, the gas pressure was reduced which results in the desorption of N_2_ molecules from the surface. The BET-specific surface area was calculated using Quantachrome Software and a DFT model was used to determine the pore size distribution of the carbon material. ICP-OES was carried out on a Varian 715-ES. Therefore, a certain amount of the sample was digested in a mixture of 2 ml sulfuric (98 wt%), 2 ml nitric (69 wt%), and 6 ml chloric (37 wt%) acids in a microwave (CEM SP-D Discover) until it became transparent. XPS was carried out on a K-Alpha™ + X-ray Photoelectron Spectrometer (XPS) System (Thermo Scientific), with a Hemispheric 180° dual-focus analyzer with a 128-channel detector and the X-ray monochromator was micro focused Al-Kα radiation. N1s spectra were fitted with eight Gauss–Lorenz (FWHM: 1.6 ± 0.05 eV, GL ratio: 0.65) and Gauss convoluted Doniach–Sunjic (FWHM: 1.45 ± 0.05 eV) functions after testing various combinations of fit parameters (Peaks 1–8). The eight peak functions were then clustered into 4 binding energy (BE) ranges and denoted as BE-range 1–4. BE-range 1 includes Peak 1 (398.1 eV) and Peak 2 (398.7 eV) covering N bonded with 2 sp^2^ carbon atoms and –C=N–C double bond motifs (*e.g.* imine N, pyridinic N, or triazinic N). BE-range 2 includes Peak 3 (399.3 eV) and Peak 4 (399.8 eV) covering sp^2^ hybridized N in metal–N coordination, OC–NH–C partial double bonds, or multiple graphitic N motifs in a single aromatic ring (*e.g.* M–N_*x*_, amides). BE-range 3 includes Peak 5 (400.7 eV), Peak 6 (401.8 eV), and Peak 7 (403 eV) covering hybridized N bonded with 1 H (in-place hydrogenated N), isolated graphitic N, out-of-plane hydrogenated/protonated N, and hydrogenated graphitic N (*e.g.* pyrrolic or protonated pyridinic and protonated graphitic N). BE-range 4 includes Peak 8 (405 eV) covering oxidized N (*e.g.* C=N–O or other oxidized N). The Gauss convoluted Doniach–Sunjic function was employed for Peak 5 (400.7 eV), Peak 6 (∼401.8 eV), and Peak 7 (∼403.0 eV) to account for the asymmetry on the high binding energy side of the major spectral peak. Fe, Ni L-edge XAS data were collected at the Innovative Station for *In Situ* Spectroscopy (ISISS) beamline of BESSY II of the Helmholtz–Zentrum Berlin at RT under ultra-high vacuum conditions.^46^ The Fe, Ni L-edge spectra were recorded in the total electron yield mode detected with a Faraday cup. STEM images and energy-dispersive X-ray (EDX) spectroscopy elemental maps were acquired with a probe-corrected (Cs-corrected for the illumination system) JEM-ARM200F NeoARM scanning transmission electron microscope (JEOL Ltd.), operated at 80 kV using a cold-FEG, equipped with a Dual-EDX-System with 2 × 100 mm^2^ detectors. Specimens were prepared by drop-casting 3 μl of the sonicated dispersion onto a lacey carbon TEM grid and dried in air. ^57^Fe Mössbauer spectroscopy was performed in transmission mode at room temperature. A CMCA-550 (WissEI) equipped with a constant electronic drive system and triangular reference wave form (Halder Electronics) combined with a ^57^Co/Rh source was used. For calibrating the velocity range the isomer shift *δ*_iso_ of the sextet lines of α-Fe foil (25 μm thick, 99.99% purity) within a velocity range of ± 8 mm s^−1^ and ± 10 mm s^−1^ was used for Fe–NC and FeNi–NC, respectively. The Mössbauer spectra were fitted with MossWinn 4.0i.

### 4.3. Electrochemical measurements of ORR activity and selectivity

Rotating-ring disk electrode (RRDE) measurements were performed to determine the ORR activity of PANI-derived non-noble metal catalysts. Therefore, 15.7 mg catalyst powder, 750 μl de-ionized water, 190 μl ethanol and 60 μl Nafion were used to prepare an ink and sonicated for 15 min. The required aliquot of the ink was dropped on a glassy carbon disk (0.2475 cm^2^) to achieve a loading of 800 μg cm^−2^. A carbon rod and a reversible hydrogen electrode (RHE) were used as the counter electrode and reference electrode, respectively. As electrolytes O_2_-saturated aqueous solutions of 0.1 M KOH and 0.1 M HClO_4_ were used. The potential range of the disk was set from 0.05 to 1.1 V *vs.* RHE in an alkaline environment, and 0 to 1.1 V *vs.* RHE for the acidic electrolyte by choosing 5 mV s^−1^ scan rate at 800, 900, 1200, 1600 and 2000 rpm. The potential for the ring was kept at 1.2 V *vs.* RHE. The collection efficiency of the RRDE electrode used was 0.37 ± 0.01.

The kinetic current density (*j*_k_), kinetic mass activity (*j*_m_), Tafel slope (mV per decade), fractional hydrogen peroxide (H_2_O_2_) yield (%) and electron transfer number are based on the following equations:

Kinetic current density (*j*_k_): combining the diffusion and kinetic controlled region, the total current can be determined using the Koutecky–Levich equation, which is expressed as follows:5
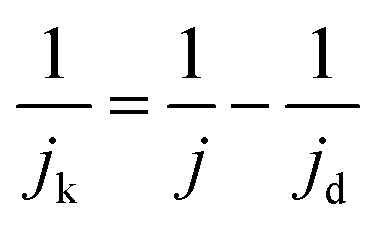
where the diffusion limiting current density *j*_d_ was chosen at potential 0.2 V *vs.* RHE.

The kinetic mass activity (*j*_m_) for the MNC catalysts in this work is defined as:6
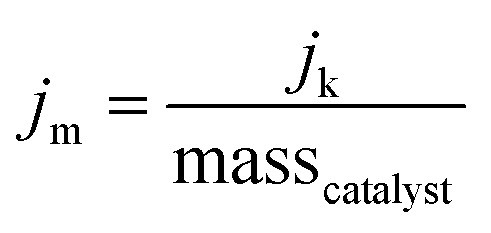
where mass_catalyst_ is the catalyst loading on the glassy carbon disc (mg cm^−2^). Because there are dramatic differences in MNC catalyst ORR activity, it is difficult to set one fixed potential for *j*_k_ and *j*_m_, as the potentials at which *j*_k_ and *j*_m_ are reported depend on the activity of MNC catalysts (which we discussed in the Results section).

Hydrogen peroxide yield (%): H_2_O_2_ (%) was calculated based on the following equation:7
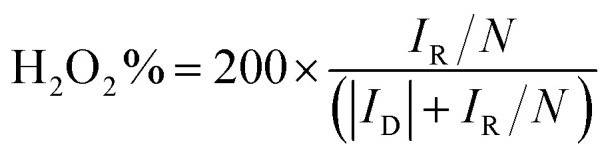


The electron transfer number was calculated based on the following equation:8
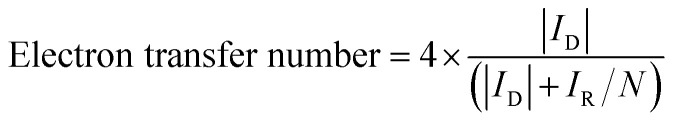
where *I*_R_ is ring current (mA), *I*_D_ is disk current (mA), and *N* is the collection efficiency.

### 4.4. CO cryo chemisorption and temperature programmed desorption (TPD)

CO cryo chemisorption experiments and TPD were performed using a Thermo Scientific TPD/R/O 1110 instrument under a 20 ml min^−1^ flow of helium as the carrier gas. 100–150 mg of catalysts was added into a U-shaped quartz reactor and underwent the three-step analysis. (i) Thermal cleaning pretreatment involved heating the sample from room temperature to 600 °C with a ramp rate of 10 °C min^−1^ under a continuous 20 sccm 5 N helium flow, followed by a hold of 15 min at 600 °C, and then followed by subsequent convective cooling back to room temperature. (ii) CO pulse chemisorption involved subsequent cooling of the pretreated catalysts to −80 °C, using a mixture of dry ice and acetone, followed by passing six consecutive 0.338 ml CO pulses, dosed at 25 min intervals, through the catalyst samples, and the amount of CO retained in the catalysts was monitored and quantified using a TCD detector. (iii) A temperature-programmed CO desorption (TPD) was performed by ramping the temperature from −80 °C up to 600 °C with a ramp rate of 10 °C min^−1^ under a continuous 20 sccm 5 N helium flow, and the CO desorption temperature peak profiles were monitored for each catalyst.

## Author contributions

F. L., P. S. and U. I. K. conceived, designed and coordinated the study. F. L. carried out the materials synthesis, characterization and electrochemical evaluations. S. W. carried out the Mössbauer spectroscopy, I. O. and S. S. carried out the STEM., S. L. and A. T. carried out the XPS, and H. W. carried out the BET measurements. J. S. and J. W. participated in the discussion of the electrochemical results section. All the authors discussed the results and commented on the manuscript. F. L. wrote the manuscript with the contribution of all the co-authors.

## Conflicts of interest

There are no conflicts to declare.

## Supplementary Material

SC-012-D0SC03280H-s001
