# Supplementary material for: Surface site density and utilization of platinum group metal (PGM)-free Fe–NC and FeNi–NC electrocatalysts for the oxygen reduction reaction
Source: Chem Sci. 2020 Oct 13;12(1):384–96. doi: 10.1039/d0sc03280h (PMC8179675; doi:10.1039/d0sc03280h)
Supplement: SC-012-D0SC03280H-s001 [file SC-012-D0SC03280H-s001.pdf]

Electronic Supplementary Material (ESI) for Chemical Science.
This journal is © The Royal Society of Chemistry 2020

Supporting Information

Surface Sites Density and Utilization of Platinum Group Metal (PGM)-free Fe-NC and FeNi-NC Electrocatalysts for the Oxygen Reduction Reaction

*Fang Luo[1], Stephan Wagner[2], Ichiro Onishi[3], Sören Selve[4], Shuang Li[5], Wen Ju[1], Huan Wang[1], Julian Steinberg[1], Arne Thomas[5], Ulrike I. Kramm[*2], Peter Strasser[*1]*

[1]The Electrochemical Catalysis, Energy and Materials Science Laboratory, Department of Chemistry, Technische Universität Berlin, Straße des 17. 10623, Berlin, Germany

[2]Department of Chemistry and Department of Materials and Earth Sciences, Catalysts and Electrocatalysts group, Technical University of Darmstadt, Otto-Berndt-Str. 3, 64287 Darmstadt, Germany

[3]JEOL Ltd, 3-1-2 Musashino, Akishima, Tokyo, Japan.

[4]Technische Universität Berlin, Center for Electron Microscopy (ZELMI), Straße des 17. Juni 135, 10623 Berlin, Germany

[5]Functional materials, Department of Chemistry, Technical Universität Berlin, Hardenbergstr. 40, 10623, Berlin, Germany

*Email: pstrasser@tu-berlin.de; kramm@ese.tu-darmstadt.de

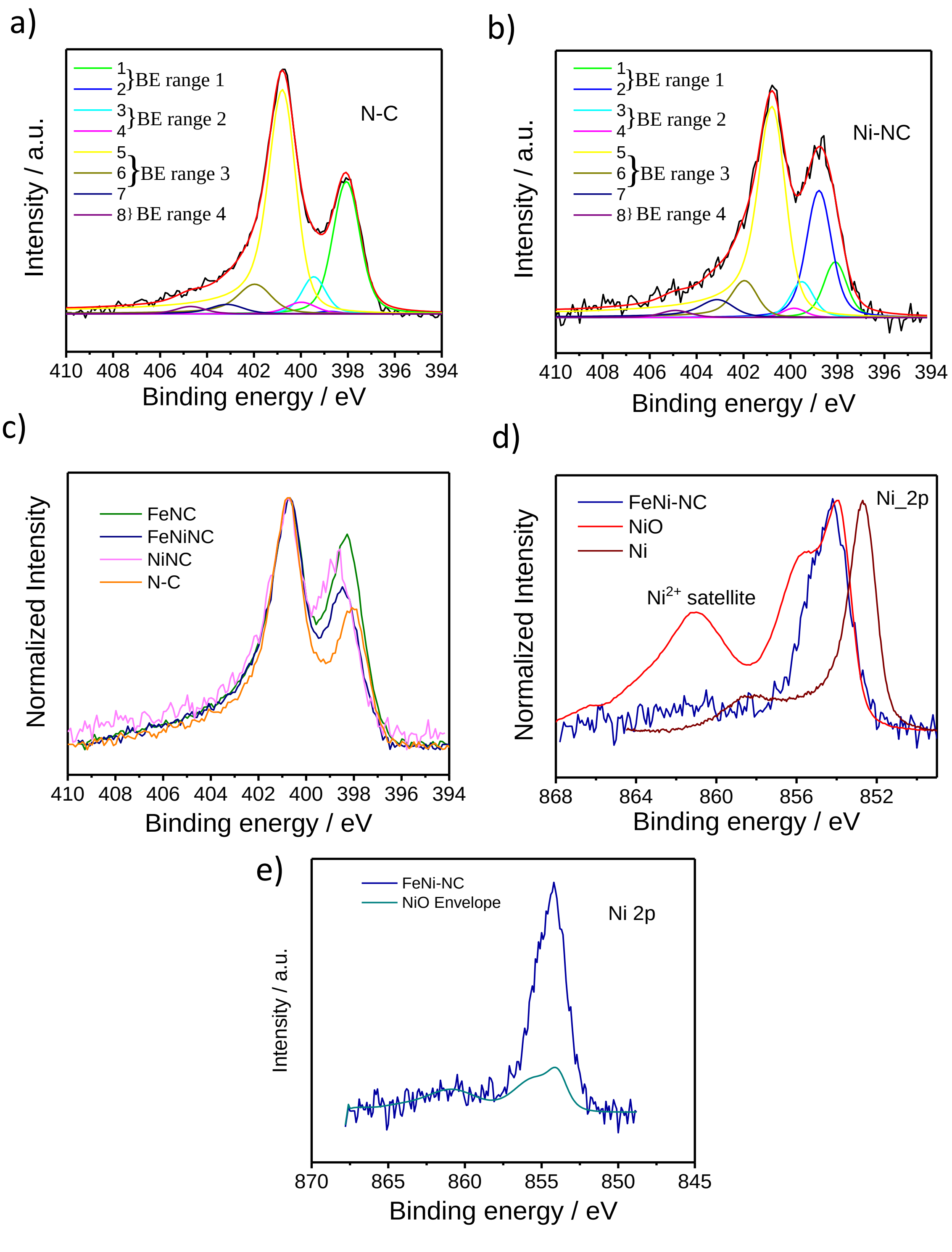

Figure S1. *XPS analyses of N-C and Ni-NC catalysts. XPS-N_{1s} spectrum of (a) N-C and (b) Ni-NC, (c)The comparison of high-resolution N 1s XPS for all the catalyst, (d) XPS-Ni_{2p} spectra of FeNi-NC, NiO and Pure Ni, (e)The comparison of experimental Ni2p spectra of FeNi-NC and expected Ni2p spectra of NiO envelope.*

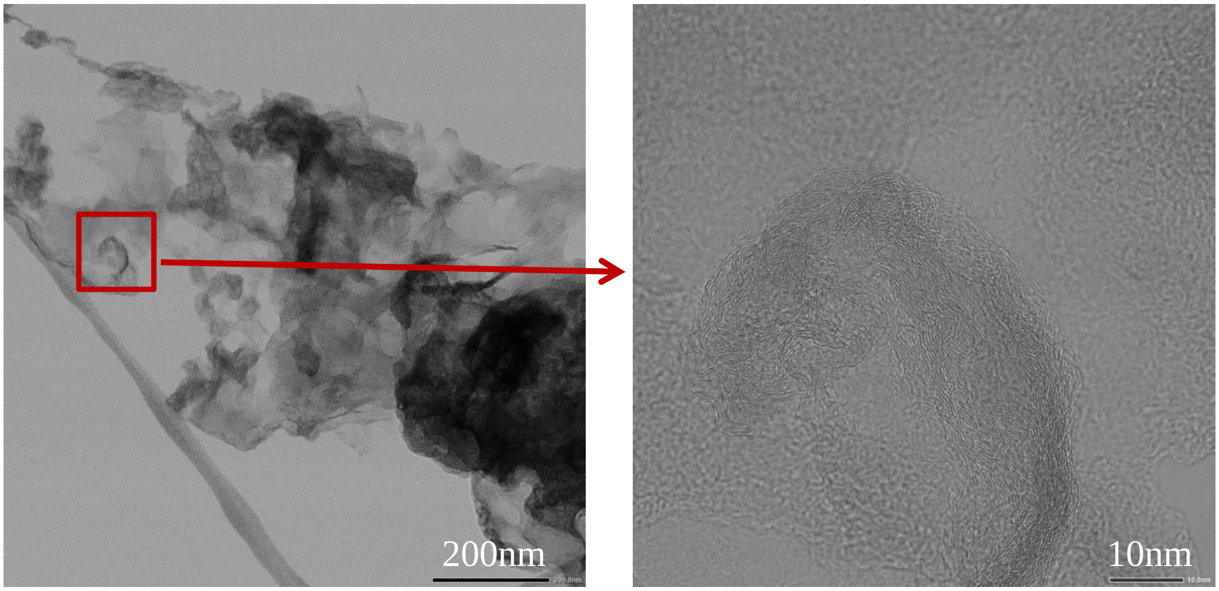

Figure S2. *STEM-BF image images of the carbon phase of FeNi-NC catalyst.*

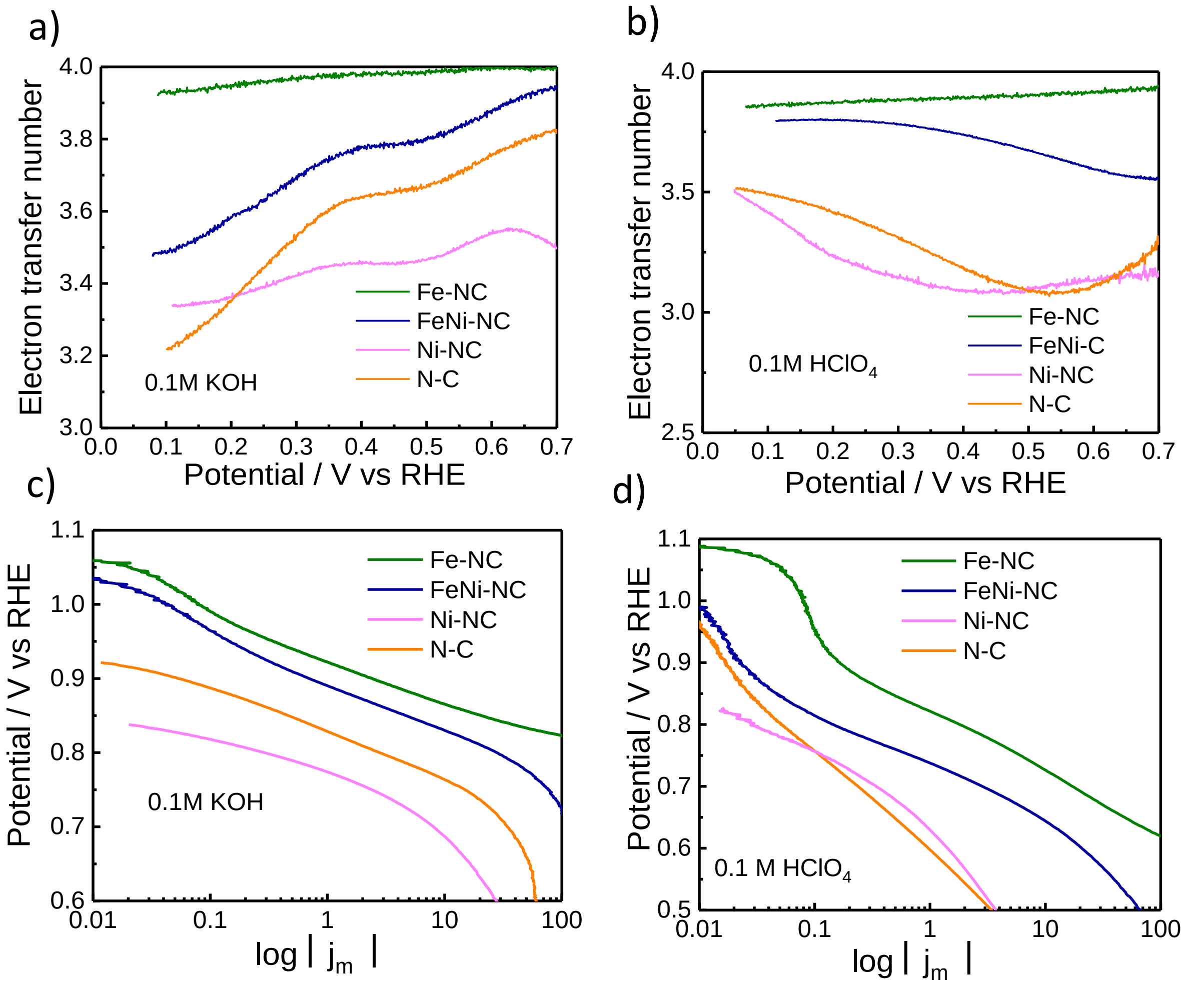

Figure S3. *Electron transfer for Fe-NC, FeNi-NC, Ni-NC and N-C in (a) alkaline and (b) acidic electrolyte. Electrochemical Tafel curves of MNC catalysts (applied E versus log|j_m| (j_m: kinetic mass activity / mA mg^{-1})) in (c) alkaline and (d) acid electrolyte.*

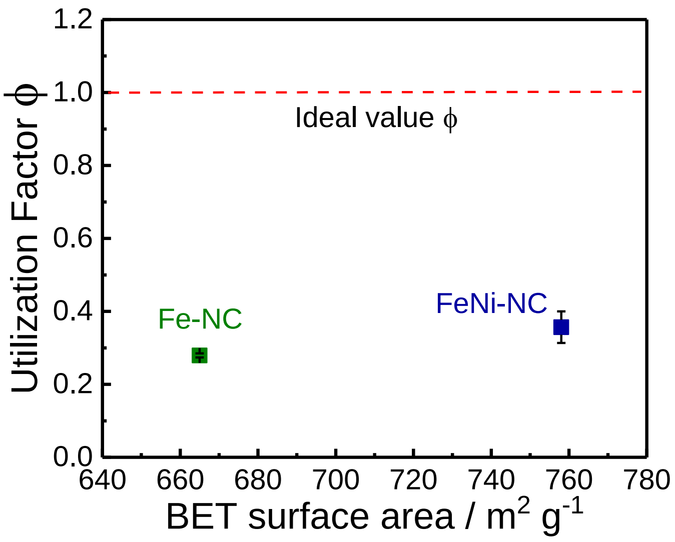

Figure S4. *The active-site utilization factor* $\phi_{SD\ surfac/bulk}$ *as a function of Brunauer-Emmett-Teller (BET) surface area.*

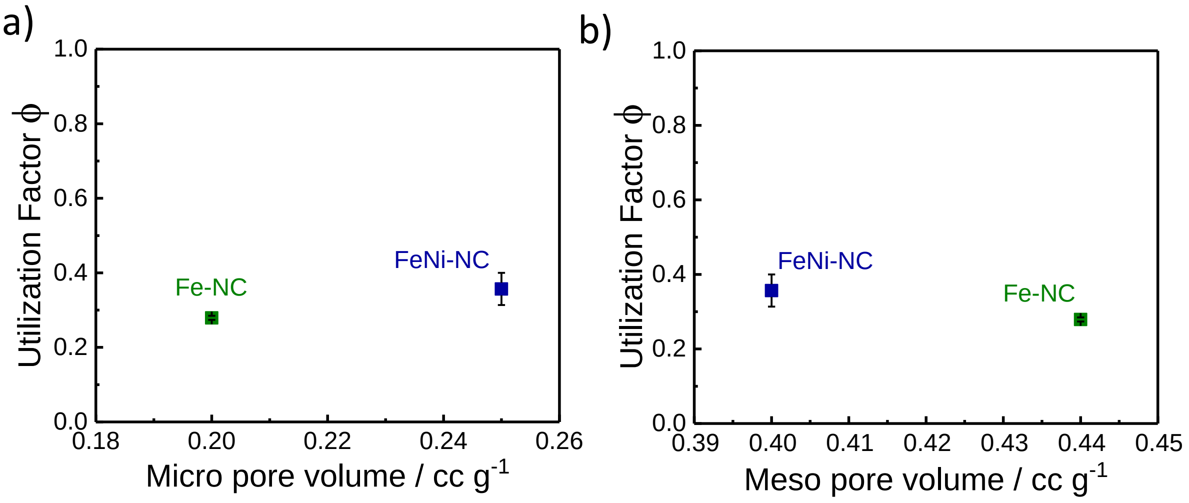

Figure S5. *The correlation of the (a) micro and (b) meso pore volumes and the utilization factors*

Supplementary Table 1. *The atomic concentrations (at.%) of N, O, C, Fe, and Ni of Fe-NC, FeNi-NC, Ni-NC and N-C catalysts from XPS.*

Sample	O	N	C	Fe	Ni
**Fe-NC**	3.8	5.9	88.9	0.8	-
**FeNi-NC**	2.5	4.3	91.7	0.4	0.4
**Ni-NC**	3.0	6.0	88.6	-	0.9
**N-C**	3.9	4.0	92.2	-	-

Supplementary Table 2. *Relative amount of nitrogen components, as obtained by fitting of the N_{1s} narrow scan XPS spectra to 8 individual N components. The latter were grouped into 4 distinct BE-ranges, due to recognized multiple possible assignment of nitrogen speciation in each of those BE ranges.*[1]

	**BE- range 1** N bonded to two sp^2 carbons, NC double bonds, -C=N-C (e.g. Imine, Pyridinic N, triazinic N) 398-399 eV / at%		**BE- range 2** sp^2 N, N-Metal coordination, OC-NH-C, multiple graphitic N in a single aromatic ring (e.g. M – Nx, Amide) 399 – 400 eV/ at%		**BE- range 3** in–plane hydrogenated N, isolated graphitic N, out-of-plane hydrogenated-N/protonated N, hydrogenated graphitic N (e.g. pyrrolic, protonated pyridinic) 400 – 403 eV / at%			**BE- range 4** oxidized N (e.g. C=N-O) / at%
**BE**	~398.1ev	~398.7ev	~399.3ev	~399.8ev	~400.7ev	~401.8ev	~403ev	~405ev
**Fe-NC**	20.6	13.6	4.4	3.6	44.9	7.4	4.3	1.3
**FeNi-NC**	15.9	11.8	2.9	3.3	50.1	8.6	5.3	2.1
**NiNC**	8.7	21.3	5.6	1.5	48.9	7.7	5.0	1.4
**N-C**	25.3	0.4	6.4	2.5	52.7	8.5	2.6	1.6

Supplementary Table 3. *Physical characterization of Fe-NC, FeNi-NC, Ni-NC and N-C catalysts*

Catalyst	Microporosity / cc g^{-1}	Mesoporosity / cc g^{-1}	Micropore Surface Area / m^2g^{-1}	BET Surface Area / m^2g^{-1}	Iron Content (ICP) / wt %	Nitrogen Content (EA) / wt %	Carbon Content (EA) / wt %
**Fe-NC**	0.20	0.45	587	665	Fe: 3.92	6.43	76.41
**FeNi-NC**	0.25	0.4	701	758	Ni: 1.74<br>Fe: 2.44	4.3	80.19
**Ni-NC**	0.05	0.22	108	238	Ni: 13.8	5.49	66.34
**N-C**	0.02	0.2	47	174	--	7.1	83.05

Supplementary Table 4. *Summary of Rotating ring disk electrode (RRDE) results in terms of mass activity-j_m at 0.85V_{RHE} for pH 13 KOH and at 0.8V_{RHE} for pH 1 $HClO_4$. All catalysts were measured in O_2-saturated electrolyte with 5 mV s^{-1} scan rate, at 1,600 rpm. Experimental errors are indicated*

**catalysts**	**0.1 M KOH, pH 13, 0.85V_{RHE}**	**0.1 M $HClO_4$, pH 1, 0.8V_{RHE}**
	j_m / mA $mg_{catalyst}^{-1}$	j_m / mA $mg_{catalyst}^{-1}$
**Fe-NC**	15.56±1.54	2.43±0.12
**FeNi-NC**	1.94±0.49	0.24±0.03
**Ni-NC**	0	0.03±0.003
**N-C**	0.50±0.06	0.05±0.004

Supplementary Table 5. *The half-wave potentials ($E_{½}$) of Fe-NC, FeNi-NC, Ni-NC and N-C catalysts from RRDE experiments*

**Samples**	**Fe-NC**	**FeNi-NC**	**Ni-NC**	**N-C**
**$E_{1/2}$ (V vs RHE) 0.1 M KOH**	0.89±0.01	0.84±0.01	0.72±0.001	0.79±0.002
**$E_{1/2}$ (V vs RHE) 0.1M $HClO_4$**	0.79±0.002	0.69±0.004	0.46±0.01	0.43±0.01

Supplementary Table 6. *CO cryo chemisorption results of Fe-NC, FeNi-NC, Ni-NC and N-C catalysts*

**Catalysts**	**n_{CO} / 10^{-6} mol g^{-1}**
**Fe-NC**	162±6
**FeNi-NC**	53±8
**Ni-NC**	0
**N-C**	0

Supplementary Table 7. *Active sites density (SD) from CO cryo chemisorption and Mössbauer spectroscopy experiments of Fe-NC and FeNi-NC catalysts*

**Catalysts**	**SD (×10^{20}) / site g^{-1}**	
	CO chemisorption	Mössbauer spectroscopy
	$SD_{surface}$	SD_{bulk}-(D1+D2)
**Fe-NC**	0.98±0.04	3.49±0.07
**FeNi-NC**	0.32±0.05	0.90±0.01

Supplementary Table 8. *The utilization factor ($\phi_{SD\ surfac/bulk}$) results of of Fe-NC and FeNi-NC catalysts*

**Catalysts**	$\phi_{SD\ surfac/bulk}$
	SD_{bulk}-(D1+D2)
**Fe-NC**	0.28
**FeNi-NC**	0.36

Supplementary Table 9. *The turn over frequency (TOF) results of Fe-NC and FeNi-NC catalysts*

**Catalysts**	**TOF / e/(s*site) KOH**	**TOF/ e/(s*site) $HCLO_4$**
**Fe-NC**	1.00	0.15
**FeNi-NC**	0.38	0.05

References

1. Artyushkova, K., Misconceptions in interpretation of nitrogen chemistry from x-ray photoelectron spectra. *Journal of Vacuum Science & Technology A*, 2020, ***38***, 031002.
